# A Review of Design and Fabrication of the Bionic Flapping Wing Micro Air Vehicles

**DOI:** 10.3390/mi10020144

**Published:** 2019-02-21

**Authors:** Chen Chen, Tianyu Zhang

**Affiliations:** College of Instrumentation & Electrical Engineering, Key Laboratory of Geophysical Exploration Equipment, Ministry of Education of China, Jilin University, Changchun 130026, China; cchen@jlu.edu.cn

**Keywords:** bionic flapping-wing micro air vehicle, aerodynamic mechanism, mechanical transmission, actuator, power electronic interface

## Abstract

Bionic flapping-wing micro air vehicles (FWMAVs) are promising for a variety of applications because of their flexibility and high mobility. This study reviews the state-of-the-art FWMAVs of various research institutes driven by electrical motor, mechanical transmission structure and “artificial muscle” material and then elaborates on the aerodynamic mechanism of micro-winged birds and insects. Owing to their low mass budget, FWMAVs require actuators with high power density from micrometer to centimeter scales. The selection and design of the mechanical transmission should be considered in parallel with the design of the power electronic interface required to drive it. Finally, power electronic topologies suitable for driving “artificial muscle” materials used in FWMAVs are stated.

## 1. Introduction

At present, there are many complex and cluttered environments that humans cannot survive in for a long time, such as glaciers, deserts, dense forests and caves. To explore these rigorous environments, flapping-wing micro air vehicles (FWMAVs) have been included in research by many scientific institutions as one of the feasible solutions. The advantages of FWMAVs are their more flexible maneuverability and more efficient aerodynamics compared with those of fixed or rotary wing air vehicles.

Remarkable achievements have been accomplished with regard to designing and optimizing the constituent subsystems of FWMAVs, including aerodynamic mechanism [1], mechanical transmission [2], actuator [3] and power electronic interface [4]. Nevertheless, several critical challenges have to be resolved urgently to increase the practical ability of FWMAVs. This manuscript presents the (a) current research progress of existing FWMAVs investigated by scientific institutions; (b) aerodynamic mechanism of FWMAVs, including birds and insects; (c) actuators composed of new material; and (d) related power electronic interface. The aforementioned literature reviews can be used as a reference by researchers.

The remainder of this paper is organized as follows: Section 2 introduces the latest achievements in FWMAVs conducted by several research institutes; Section 3 presents the aerodynamic mechanism of FWMAVs, mechanical transmissions and various types of actuators and related power electronic interfaces; and finally, Section 4 discusses the conclusions and the future research prospects for FWMAVs.

## 2. FWMAVs with Different Actuation Mechanisms

Research on FWMAVs can be divided into three types according to the driving methods, including electrical motor, mechanical transmission and “artificial muscle” material.

### 2.1. Electrical Motor-Driven Method

Many institutions investigate electrical motor-driven FWMAVs. Although the electrical motor-driven method does not deliver excellent performance in terms of energy efficiency, it remains the first choice for most FWMAVs because of its mature application scheme and low manufacturing cost. 

“Microbat” [5] was developed by the California Institute of Technology in the US in 2002. It was the first FWMAV driven by electrical motor. This aerial vehicle achieves self-contained autonomous flight by mimicking the morphological properties of flexible bat wings with a mass of 12.5 g, wingspan of 25 cm and more than 42 s flight time. The aircraft flew under radio control, where the pilot could control left and right turns, pitching angle and motor on/off. During this flight right and left turns were commanded and aircraft responded appropriately. The flight duration was mainly limited by the power system and vehicle’s weight.

“Hummingbird” [6] is an FWMAV with a camera and autonomous control support, launched by AeroVironment, USA, in 2011. The aerial vehicle has a mass of 19 g, a wingspan of 16.5 cm and a flight time of 4 min. As it does not completely draw on the design of bionics, it still has a certain discrepancy with actual hummingbirds, leading to its poor performance in propulsion efficiency and motion sensitivity aspects. The Hummingbird is equipped to carry its own energy source with it while flying. The wings attached to the NAV will help to rotate and turn towards any angle and position as directed by the controlling crew. The flapping wings allow the NAV to control its attitude during flight. The vehicle can be controlled remotely from a distance of up to one kilometer. Nevertheless, “Hummingbird” remains one of the FWMAVs that have already been put into practical use at high level.

“Phoenix” [7] was manufactured by the Massachusetts Institute of Technology, USA, in 2011. A huge lift is generated by utilizing the huge wings to support an airframe weighing 1200 g. “Phoenix” combines a mechanical, pressure-independent regulator with a high-speed position/airflow controller to meet the unique requirements of airflow control. However, because of its large extra energy requirement, the vehicle should be started manually and can fly only for a short distance.

“H^2^bird” [8], with a mass of 13.6 g and a wingspan of 26.5 cm, was investigated by the University of California, Berkeley, USA, in 2014. Linear piece-wise affine modeling of segments of flight conditions within a maneuver was used as an effective method for determining transition points between hybrid controllers. The controllers was stored on-board to enable autonomous navigation or obstacle avoidance by picking maneuvers applicable to an observed situation. Since the models are linear, the computational overhead for onboard look-ahead simulation or computation of feedback controllers is reduced relative to complex nonlinear models.

An experimental FWMAV with an integrated autopilot was fabricated by the University of Arizona, USA, in 2009 [9]. It weighs 248 g, has a wingspan of 74 cm and maintains 7 min flight endurance. The autonomous ornithopter is a test bench to investigate the aerodynamic parameters of a flapping aerial vehicle, such as aerodynamic forces, kinematics and automatic controls. The ornithopter competition involves building the smallest radio-controlled ornithopter that can fly the most laps around a pylon course in two minutes. It is believed that the overall quality of flight plan tracking can be improved by further optimization of the flight control system. 

Eagle flight simulator model was designed by the University of Maryland, USA, in 2008 [10]; it has a weight of 425 g and a wingspan of 107 cm. Researchers used the aircraft to obtain and verify relevant aerodynamic parameters during flight. In 2013, Gerdes et al. fabricated the pioneering flight of “Robo Raven” which is a major breakthrough for micro air vehicles [11]. “Robo Raven” has a wingspan of 150 cm and a minimum weight of 690 g. It was the first demonstration of a bird-inspired platform doing outdoor aerobatics using independently actuated and controlled wings. Independent wing control has the potential to provide a greater flight envelope.

“Smart Bird” [12] is a bionic FWMAV developed by Festo Company in Germany in 2015. It is an example of bio-mimicking seagull flight. The latest smart bird has a mass of 450 g, a wingspan of 50 cm and can achieve up to 80% aerodynamic efficiency when flying on a circular trajectory/bound orbit. A powerful microcontroller calculates the optimal setting of two servo motors, which adjust the torsion of each wing. The flapping movement and the torsion are synchronized by three Hall sensors, which determine the absolute position of the motor for the flapping movement. However, precision control cannot be easily reached.

“DelFly” [13,14,15,16] was developed by Delft University of Technology in the Netherlands in 2005. A coaxial four-wing FWMAV is achieved by mimicking the flight mode of beetles. The first generation “DelFly” has a mass of 21 g and a wingspan of 50 cm and its follow-up generation achieves a low mass of 3.07 g and a short wingspan of 10 cm. In addition, “DelFly” has single degree of freedom motor-driven flapping wings for generating thrust. The control moments are generated by actuated control surfaces on the tail. The tail actuation typically consists of a rudder and an elevator and can be used for changing the MAV’s direction, height or velocity. Since the tail is relatively large, it dampens the body dynamics sufficiently to make this type of FWMAV passively stable.

The motorized insect-mimicking FWMAV [17], designed by Korean Konkuk University in 2015, has a wingspan of 12.5 cm and weighs 7.36 g with batteries and power control installed. This work has successfully demonstrated a micro aerial vehicle that can stably takeoff with initial stability. An uncontrolled takeoff test successfully demonstrated that the FW-MAV possesses initial pitching stability for takeoff.

“Bat Bot” [18] was fabricated by the California Institute of Technology and the University of Illinois at Urbana-Champaign in 2017. It weighs 93 g and is shaped like a bat with a wingspan of approximately 1 ft. To mimic the morphological properties of bats, researchers used custom-made silicon skin and articulated morphing wings. The flight mechanism involves several different types of joints that interlock the bones and muscles to one another, creating a musculoskeletal system that is capable of movement in more than 40 rotational directions.

A flapping twin-wing robot of the size of hummingbird (Colibri in French), actively stabilized in pitch and roll and capable of hovering, was constructed by Roshanbin, A. et al. at the Universite’ Libre de Bruxelles in 2017 [19]. The prototype has a total mass of 22 g and a wing span of 21 cm. The controller was tuned for the linearized, cycle-averaged model and can stalely control the flight in 4 DOF by altering 4 parameters: the flapping frequency, the difference between the left and right wing flapping amplitude and, independently, the left and right wing offset. The robot has demonstrated successful hovering flights with a 15–20 s onboard battery for flight autonomy. A better control of lateral direction flight can be searched in the future.

The FWMAV, the Golden Snitch, developed by Hsiao et al. at Tamkang University in 2012 [20]. It is an 8 g weight and 20 cm wingspan aircraft, including the fuselage, flapping wings, tail wings, battery, motor and gear system. The flapping wings are driven by a motor with a four bar linkage system. By adjusting the lengths of the four bars, various stroke angles can be achieved. Due to the limited payload-carrying capability, the P control architecture was modified so that automatic control of flight altitude of a flapping-wing MAV fewer than 10 g.

Wasp AE, developed by the Aerovironment company, U.S., is the all-environment version of FWMAV [21]. With special design considerations for maritime, Wasp AE delivers exceptional features of superior imagery, increased endurance and ease of use. The commercial vehicle has a total mass of 1300 g and wing span of 108 cm. It can be operated manually or programmed for autonomous operation, utilizing the system’s advanced avionics and precise GPS navigation.

Electrical motor-driven FWMAVs have the advantages of low visual and low acoustic signatures but have only short endurance flight. To enhance their flight endurance, an increase in the mass of the onboard battery is required. However, this increase adds to the overall weight of the FWMAV; in turn, more energy is consumed, especially during the ascent and hover stages. A prominent progress in increasing the FWMAV’s endurance can be achieved using a micro combustion engine as power supply but it will increase noise, resulting in poor concealment. Hence, the problem of flight endurance versus weight is a particular challenge because the benefits of flapping-wing flight are most significant at small scales or low masses.

### 2.2. Mechanical Transmission-Driven Method

**Figure 1:**
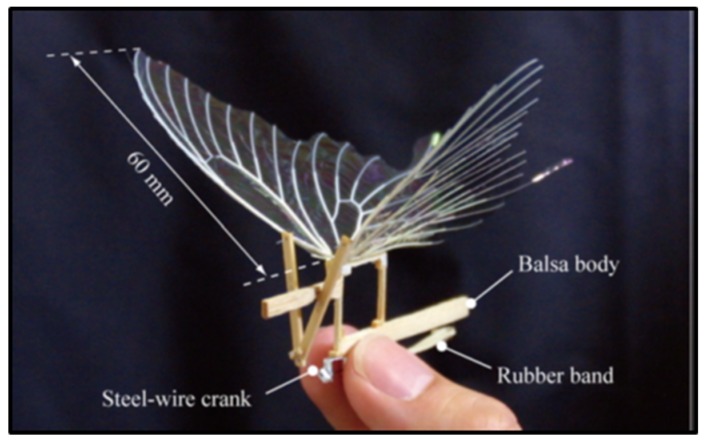
Artificial butterfly driven by machinery.

Another means of realizing ultra-light FWMAV is by using mechanical transmission-driven method. The “artificial butterfly” [22] shown in Figure 1 was constructed by the University of Tokyo, Japan, in 2010 using a butterfly-like crank-driven wing body with a flight time of only a few seconds. The flight of “artificial butterfly” is realized with simple flapping, requiring little feedback control of the feathering angle. Even, the stable forward flight could be realized without active feathering or feedback control of the wing motion

**Figure 2:**
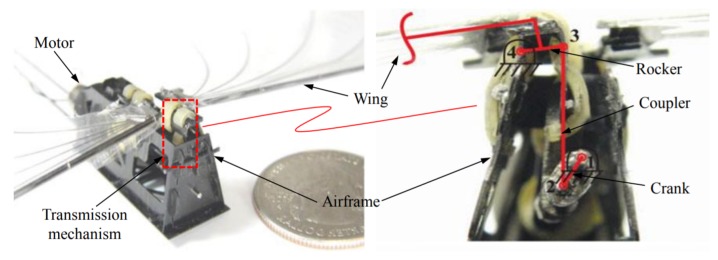
The prototype of the Harvard University’s FWMAV and its four-bar transmission mechanism.

Additionally, Sahai et al. [23] attempted to integrate flexural hinges into a four-bar compliant flapping transmission for FWMAV with approximately 3 g of weight, as shown in Figure 2.

A distinguishing feature of the mechanism is using rubber-based flexures in two of its joints (joints 3 and 4). According to the experiment, not only did compliant mechanism save up to 20% of the input power and 1% of the weight but also produced more thrust. 

**Figure 3:**
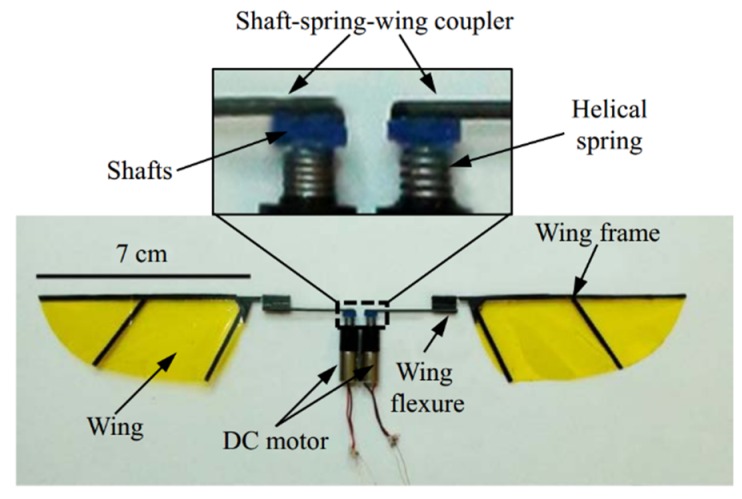
The prototype of a motor-driven flapping-wing FWMAV.

Coil Springs have also been directly coupled with DC motors for directly driving flapping wings toward resonance. Campolo et al. [24] presented a proof-of-concept flapping-wing micro aerial, shown in Figure 3. 

The prototype consists of two brushed DC motors, two 7 cm length wings, two helical springs and two shaft-spring-wing couplers. The pair of small helical springs is treated as the compliant structures for energy storage and recovery. The two separated DC motors can drive the individual wing to resonate, respectively. Experiments demonstrated the prototype can successfully lift off and the maximum lift-to-weight ratio can be achieved at the flapping frequency 10 Hz using controlling torques. 

The traditional electrical motor- and mechanical transmission-driven methods of FWMAVs have problems of low driving efficiency and large power losses at low Reynolds number during flight. Thus, initiative ideas using novel driving methods are proposed to resolve the described challenges.

### 2.3. “Artificial Muscle” Material-Driven Method

To improve flapping frequency and aerodynamic efficiency, some scientific research institutions suggested using “artificial muscles” as a new driving actuator for FWMAV instead of electrical motor and machinery. These new materials have also been applied to some present robotic applications, providing novel ideas for FWMAVs.

In 2010, the Korean Academy of Science and Technology developed a bionic crawler robot with thermal material based on the thermoelectric stretching effect [25]. In 2014, the Massachusetts Institute of Technology in the US developed a circular closed-chain robot with shape-memory material that depends on the temperature memory effect [26]. The exploration of these new materials provides alternative options and avenues for the actuator of FWMAVs. Although the mechanical deformation and energy conversion efficiency of new materials are excellent, the maximum response frequency is less than 5 Hz, which means it fails to meet the requirements of folding ratio of flapping wings. In 2013, the University of Maryland in the US developed the electrostatic material bounce robot [27] and in 2007, Sungkyunkwan University in Korea fabricated an insect animal robot [28] that has better performance in frequency response. However, due to the limitations of inherent electrical characteristics, mechanical deformation was too little to meet the large physical transformation requirement from FWMAVs. 

**Figure 4:**
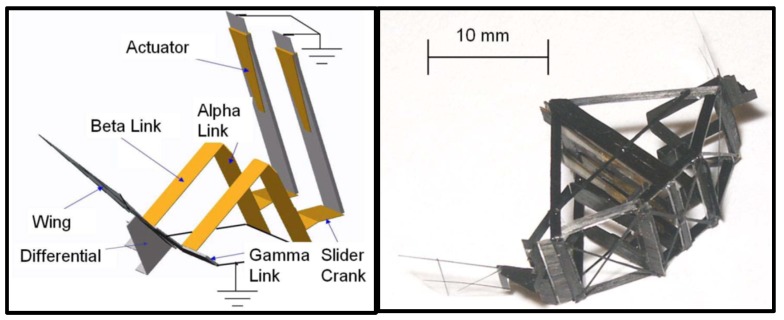
FWMAV with four degrees-of-freedom.

In addition, the University of California, Berkeley, developed a 25 mm (wingtip-to-wingtip) FWMAV capable of realizing sustained autonomous flight in 2007 [29]. Figure 4 shows the mentioned micromechanical flying insect, with four degrees-of-freedom, weighing approximately 100 mg, excluding battery or electronic devices. The main mechanical transmission component, namely, thorax, consists of two four-bar mechanisms that amplify and convert the mechanical motion into wing flapping and rotation. The biologically inspired system architecture results in a hierarchical structure of different control methodologies, which give the possibility to plan complex missions from a sequence of simple flight modes and maneuvers.

**Figure 5:**
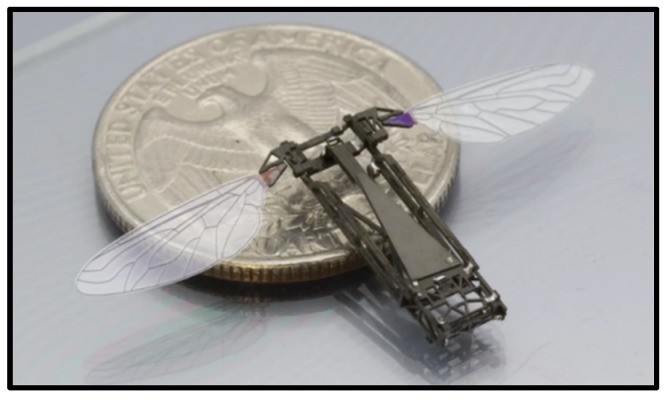
“Robobee,” an FWMAV driven by a new “artificial muscle” material developed by Harvard University.

In 2013, Harvard University proposed the use of a piezoelectric bimorph material as actuator to generate mechanical deformation using inverse piezoelectric effect [30,31,32] (Figure 5). This lift-enhancing design of mimicking the flapping mechanism of a fly’s 2 cm wingspan enabled the 80 mg FWMAV to fly autonomously. The Robobee was fitted with various individual sensors for onboard feedback. Pitch and yaw control of the RoboBee using an onboard magnetometer was presented with the robot constrained to rotate only about its principal axes. The integration of a MEMS gyroscope onto the RoboBee to provide attitude feedback in flight. However, it only worked with the connection from an external battery power supply.

The characteristics of the above FWMAVs in terms of driving method are shown in Table 1.

In conclusion, the FWMAV driven by electrical motor method is the most successful and widely used because of its high maturity, low cost and wide application in the field. Currently, the mechanical transmission-driven FWMAV is only utilized for the experimental verification of the aerodynamic model, which is not practical. Although “artificial muscle” material-driven FWMAV is in the preliminary stage, it has wide application prospects and important research significance.

In the near future, FWMAVs will evolve to become ultra-compact in size, super light and will have longer flight duration. Thus, the challenges of investigating aerodynamic mechanism, transmission mechanism and power electronic interface should be resolved.

## 3. Aerodynamic Mechanism Bases

Unlike the fixed-wing and rotary-wing aerial vehicles, the body of an FWMAV is mainly constructed based on bionics inspired by birds and insects. A flutter cycle can be divided into two stages: lower flap and upper flap. The wings are twisted quickly during the transition between the lower and upper flaps and start to flip over at the end of each stroke. The aerodynamic basis of insect flight can be divided into four types.

1. Delayed stall mechanism. For an in-depth study on the aerodynamic mechanism of flapping wings of insects, see that conducted by biologists C. P. Ellington and C. van den Ber et al. on insect behavior [33]. They used scaled-up model of hawkmoth wings for experiments. The front edge of the hawkmoth wing was equipped with a smoke-releasing device and a high-speed camera to record the changing formation of the air flow of its wings during flapping. The study indicated that the large lift produced by the hawkmoth’s wings during flapping is due to the presence of delayed stalls. The angle of attack is much larger than the conventionally critical angle of attack, a difference that cannot be explained by classically aerodynamic principles. However, the experiment revealed that the formation of a vortex of circulating air flow at the leading edge is caused by the rapid movement of the wings. A low-pressure area will be generated because the vortex is located above the wings. Thus, generating a large lift force is beneficial. The observed phenomenon is consistent with the basic theoretical calculation, which is in line with the study of Liu H. [34].

**Figure 6:**
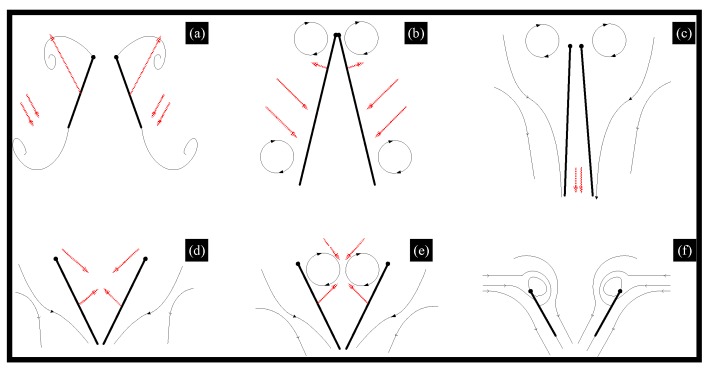
Clap-and-fling mechanism.

Clap-and-fling mechanism. Weis-Fogh discovered a mechanism of lift generation when he observed wasp flight, that is, the wings are folded back (clap) and then quickly opened (fling) before the next incitement [35] (Figure 6). The aerodynamic mechanism creates a discrete vortex at the wingtip and results in a big lift. Weis-Fogh named it the clap-and-fling mechanism, which explains the generation of large lift coefficients by insects when hovering.

As shown in Figure 4, the clap and fling mechanism consists of two phases: the first one, the leading edges of both wings are clapped together at the end of the upstroke (from (a) to (c)) and the second one, the wings rotate around their trailing edges, thus flinging apart (from (d) to (f)). During the first “fling” phase, the fling motion is produced by a rotation of the wings about the common trailing edge, a pair of large leading edge vortices are formed. During the second “fling” phase, air flows around the leading edge of each wing which creates a bound vortex on each wing acting as the starting vortex for the opposite wing. This allows a rapid buildup of circulation as well as an increase in total lift production.

2. Rotational circulation mechanism. Dickinson M. H. et al. completed the experiment using a mechanical device to obtain the equations of the wings’ flapping motion captured by the camera [36,37,38]. They simulated the movement of insect wings by driving the model wings placed in the cylinder and utilized a sensor to measure the lift and drag acting on the airfoil. As a result, they found that the translational force generated by the wing attack was not sufficient, whereas they discovered rotational circulation mechanism generated more lift, usually two to three times the chord length. The theory of rotational circulation mechanism is that the wing of the fruit fly generates a reverse vortex when the wings are flapping forward at the end. So the airflow velocity above the fly is faster than at the bottom, forming a pressure difference and producing enough lift.
3. Added mass effect mechanism. This is known to play a substantial role in defining the hydrodynamic forces acting on moving bodies because the movement of the surrounding fluid requires an additional force over and above what is necessary to accelerate the body itself [39]. Moreover, the mechanism was later devoted mostly to fast oscillating motions in view of flutter and stability studies. Andro J. Y. and Jacqin L. recently analyzed the added mass effect on a harmonically heaving airfoil by using 2-D direct numerical simulations [40]. Basing on previous studies, Giesing J. P. developed an unsteady panel method for calculating the forces acting on an airfoil executing arbitrary motions and calculated the added mass coefficients [41]. A fairly good agreement was found between the numerical and analytical values of the coefficients. Although many researchers made some achievements, the theoretical model to explain a variety of complex parameters still requires further improvement.

## 4. Transmission Mechanism Policies

**Figure 7:**
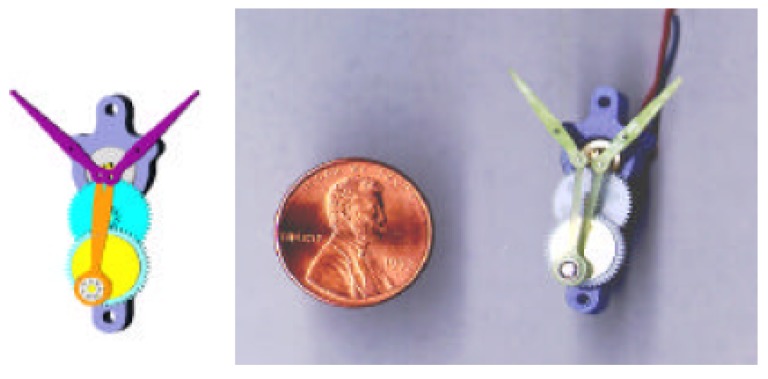
Internal transmission structure of Microbat.

Mechanical transmissions, such as electrical motors and smart materials, are investigated and designed based on the former discovered aerodynamic mechanism bases. Electrical motors are reliable, versatile, low cost and easily purchasable in the commercial market. Most of the FWMAVs described in Section 2 are driven by electrical motors; the first one is Microbat in 2001, whose transmission structure is shown in Figure 7 [5]. The rotation of the electrical motor drives the gear to actuate FWMAVs and is the common transmission principle in electrical motor-driven micro aircraft. However, mechanical transmissions have individualized designs based on their characteristics.

New smart materials have also emerged that have attracted widespread attention. If the size of the flying robot is reduced to millimeter levels, then the efficiency of the conventional electrical motor will be reduced dramatically. Therefore, various smart actuators are an optimal alternative choice for FWMAVs. Smart actuators are micro-mechanical devices that use artificial materials to generate deformation [42]. Table 2 shows the overview characteristics of smart actuators, such as strain, stress, elastic energy density, efficiency and response speed [43].

As illustrated in the table, shape memory alloy (SMA), shape memory polymer (SMP), electro-chemo-mechanical conducting polymer (EMCP), thermal polymer and mechanochemical polymer (MCP) are capable of large free strain and high resistance but have slow response and limited efficiency, which make them unsuitable for driving FWMAV. By contrast, piezoelectric actuators exhibit relatively low free strain. They have the ability to produce very high blocking forces and more efficient sensitivity. Owing to speed requirements, piezoelectric, dielectric elastomers (DEAs), electrostatics and electromagnetic actuators are effective alternatives to micro bionic flapping wing aerial vehicles.

**Figure 8:**
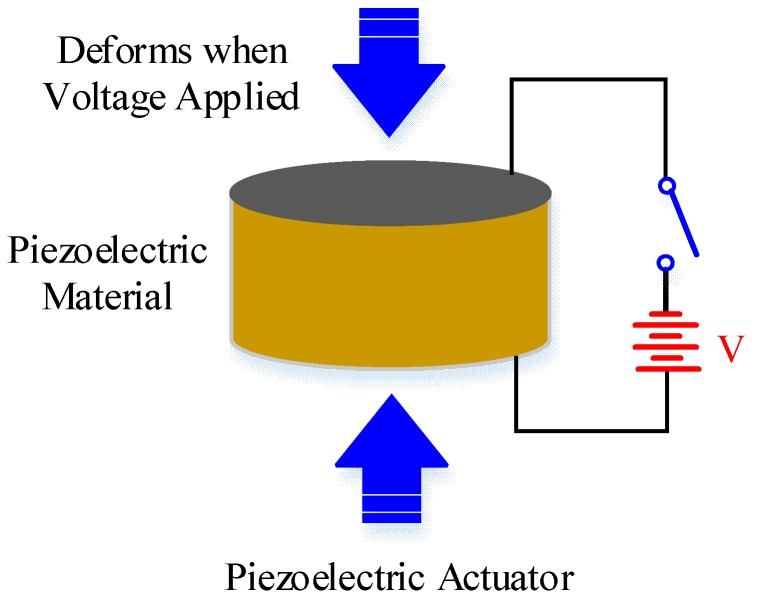
Typical example of piezoelectric actuators.

1. Piezoelectric actuators: Piezoelectric actuators are devices that use inverse piezoelectric effects [44] (Figure 8). The drive voltage of a piezoelectric actuator is typically in the range of a few tens to several hundreds of volts. The operating voltage of piezoceramic stack actuators is realized by stacking monolithic multilayer elements in the range of 60–200 V and a higher required voltage of approximately 1000 V for discrete stack actuators. When in conjunction with a mechanical transmission, the actuator is capable of enhanced stroke amplitude and reciprocating motion for flapping flight [45,46]. In addition, piezoelectric actuators have high displacement, fast response [47,48] and high efficiency at high deformation frequency [49]. Therefore, piezoelectric materials are an optimal choice for use as an actuator in FWMAVs.

At Harvard University, Wood et al. [30,45,48,49,50,51,52,53] conducted an in-depth study on an insect-scale flutter robot called RoboBee that uses a piezoelectric actuator. RoboBee was the first insect-sized robot with the ability to fly.

**Figure 9:**
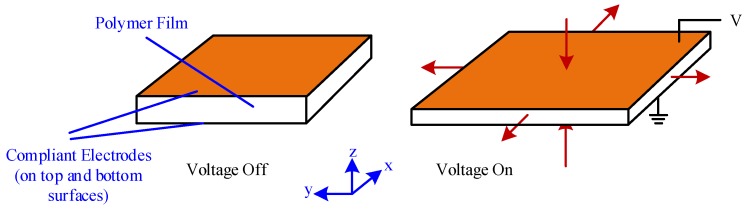
Working principle of DEA.

**Figure 10:**
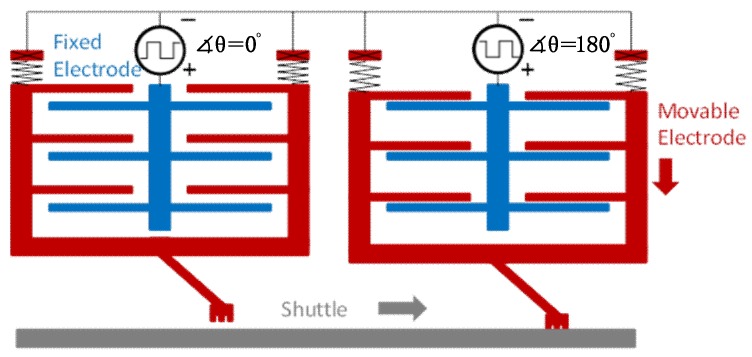
Electrostatic inchworm motor.

**Figure 11:**
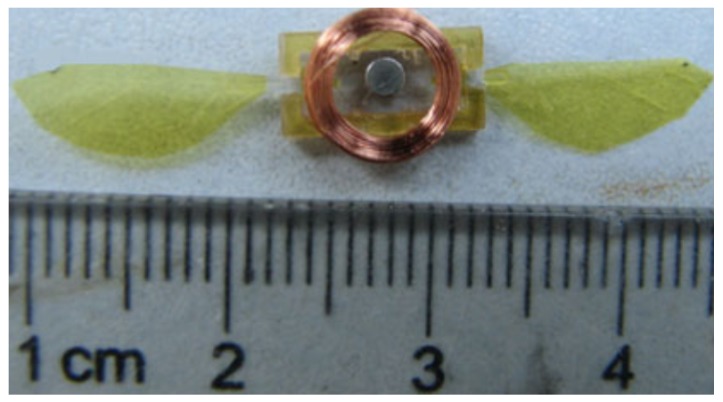
A FWMAV using electromagnetic actuator.

2. Dielectric elastomers: DEA is polymer material with flexible electrodes that have a large electromechanical response to the applied electric field (Figure 9) [53,54]. DEA typically operates at very high voltages (about 1–10 kV) with an electric field of approximately 100 MV/m and produces large strain at high working density [55,56]. In reference [57], DEA was used to drive approximately 15 g of FWMAV that extends the limitation of the artificial muscle to the level of energy required for a heavyweight aerial vehicle. However, the application is limited by the challenge of a high electric field requirement in the development of DEA.
3. Electrostatic elastomer: Electrostatic and piezoelectric actuators both offer efficient compliant actuation and are capable of providing high working densities [48]. Piezoelectric bimorph actuators have been successfully implemented for centimeter-scale robots [47] but the performance of thin film required by millimeter-scale robots deteriorates [58]. To make up for this disadvantage, electrostatic actuators are generally fabricated in chip level with Microelectromechanical Systems (MEMS) technique, which provide an excellent choice for mobile microrobots (Figure 10) [59].
4. Electromagnetic actuators: Electromagnetic actuators convert electrical energy to mechanical energy and vice versa by using electromagnetic mechanical principles. Electromagnetic actuators exhibit good performance owing to their quick response, simple structure, easy control and low voltage requirement from 0 to 24 V [60,61]. Electromagnetic actuators mainly consist of an electromagnetic coil, a permanent magnet rotor and a “virtual spring” magnet pair. Deng et al. [62] recently used a 2.6 g electromagnetic actuator to drive a FWMAV with wing-beat frequency, as shown in Figure 11.

At present, most insect-scale FWMAVs are driven by piezoelectric actuators [3,4,31,47]. Although attempts were made to use electromagnetic actuators and insulative elastomer actuators (dielectric and electrostatic actuators) for driving FWMAVs, no report indicates that aerial vehicle prototypes can be lifted successfully.

## 5. Power Electronic Interfaces

Most compact energy sources potentially suitable for FWMAV applications, such as supercapacitors [63], solar cells [64] and fuel cells [65], generate output lower than 5V. At present, conventional batteries are the only commercially available technology that is appropriate for FWMAV. The actuators mentioned earlier are classified into two actuation modes. The first is the current mode, which requires high current and relatively low voltages and corresponds to SMA, SMP, EMCP, thermal actuator and MCP. The second is voltage mode, which requires high voltages and relatively low currents and corresponds to piezoelectric, DEA, electrostatic and electromagnetic actuators.

The use of the above actuators requires a power electronic interface with high power efficiency and density to transfer energy from power source to actuator. The power electronic interface generally consists of a power stage, which regulates the voltage of the energy sources to the required level and a drive stage, which uses the output voltage to generate a time-varying signal applied on the smart actuator. This section illustrates a potential solution (not currently used) for the power electronic interface of both current- and voltage-mode actuators.

### 5.1. Power Electronic Interfaces for Current-Mode Actuators

Current-mode actuators rely on high current to raise the temperature of the active material through resistive heating. Generally, the voltage delivered to the actuator is lower than the energy source voltage. 

One of the simplest ways to convert the energy source voltage to the required low level is to use the conventional buck converter. Two alternative schemes are described to realize low voltage with compact package: n-stage cascade buck converter [66] and tapped inductor buck converter [67]. 

**Figure 12:**
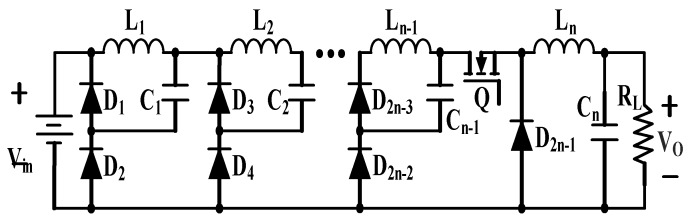
N-stage cascade buck converter.

The scheme consisting of an n-stage cascade combination of buck converter with single active switch is shown in Figure 12. 

This kind of converter requires an active power MOSFET and 2n-1 passive diodes and can be utilized only when the required number of stages is not very large. Otherwise, the whole conversion efficiency will deteriorate due to the parasitic losses of components. 

**Figure 13:**
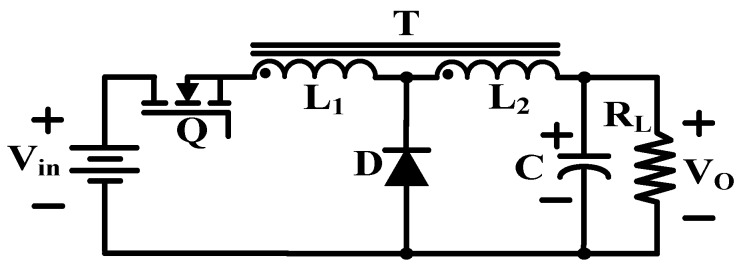
Tapped inductor buck converter.

Another feasible topology named tapped inductor buck converter is presented in Figure 13. 

This circuit utilizes a tapped inductor operated by one active switch to achieve high step-up ratio with the square of turns ratio between the primary and secondary windings.

The circuit architecture of the two topologies reveals that the latter topology requires fewer components than the former but since no tapped inductor is commercially available on the market, the circuit manufacturing technology is the main challenge for the latter topology. For the former one, the larger the output power, the higher the efficiency is subject to the exponential distribution. In addition, the upper efficiency is limited by the number of stages, while the latter is more efficient.

### 5.2. Power Electronic Interfaces for Voltage-Mode Actuators

The power electronic interfaces should be able to convert the low input voltage of a lithium battery to a high voltage signal that drives the piezoelectric or dielectric actuator [68]. Unlike current-mode actuators, voltage-mode actuators require up to several hundred volts. Recovering unused energy from the actuators is also another challenge for power electronic interfaces because only a portion of the input electrical energy is converted into a mechanical deformation of the actuators. Owing to losses in the passive inductor and active switch, as well as a very high switching frequency, the conventional boost converter becomes impractical to resolve the above challenges. Five alternative electrical interfaces are presented to achieve high voltages in a compact package: hybrid voltage multiplier boost converter, tapped inductor boost converter, cascade boost converter, high conversion ratio boost converter and power amplifier using a piezoelectric transformer (PT) [69].

**Figure 14:**
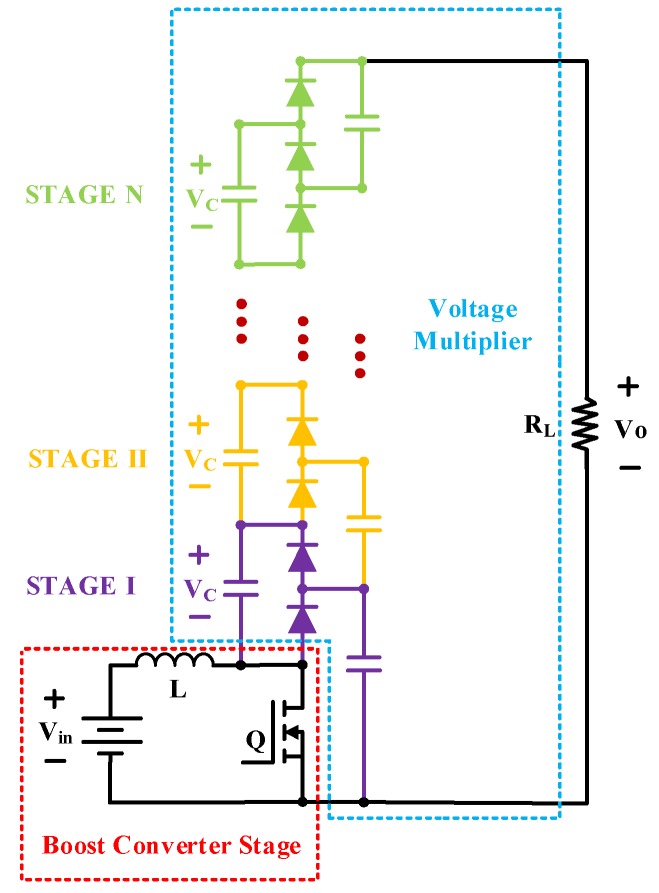
Hybrid voltage multiplier boost converter.

A hybrid topology consisting of a conventional boost converter cascaded with a switched-capacitor charge pump circuit, as shown in Figure 14, has been considered previously to drive piezoelectric actuators [70] and electrostatic MEMS devices [71]. It is an n-level DC–DC converter using one switch, 2n + 1 diodes and 2n capacitors. Operating in a regime of high efficiency, the boost converter stage provides a moderate boost to the input voltage, while its pulsed output naturally charges up the capacitor ladder through the diodes. The charge pump circuit multiplies the boost converter’s output voltage, ideally by a factor equal to the number of charge pump stages. The output power is limited by the size of the charge pump capacitors and the maximum output power of the boost converter. 

**Figure 15:**
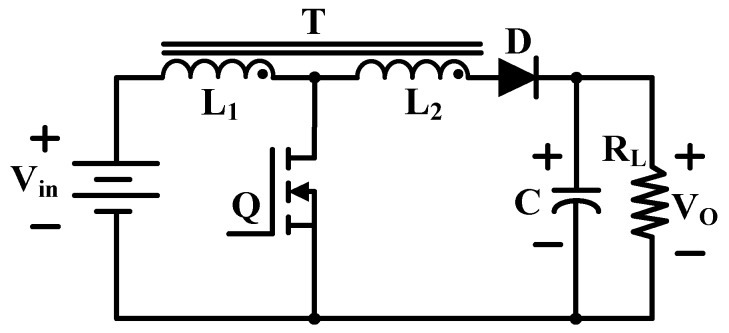
Tapped inductor boost convertor.

As shown in Figure 15, replacing the inductor in a classical boost converter with a tapped inductor results in a combination of boost and flyback converter topology, named the tapped inductor boost converter [72]. The voltage gain of this converter is greatly improved, which depends on the switching duty cycle and the transformer turns ratio.

**Figure 16:**
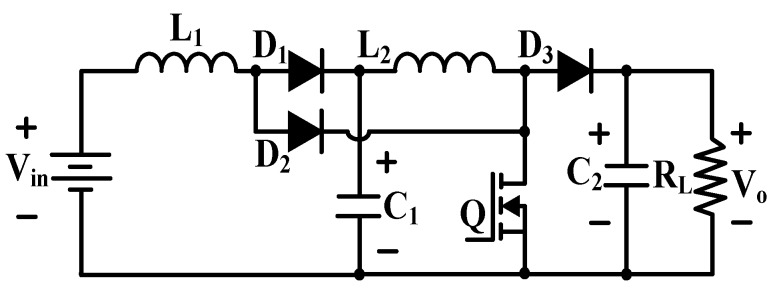
Cascade boost converter.

Super voltage boost technology is widely used in electronic converter design to increase the voltage transmission gain. Despite the high complexity of the converter, the super voltage boost converter can generate output voltages that are related to the geometric progression of the cascaded circuit [73,74,75]. Two-stage cascade boost converter is cascaded by conventional boost converters, as shown in Figure 16. It can sufficiently meet the high driving voltage requirement of piezoelectric actuators because of its high gain performance.

**Figure 17:**
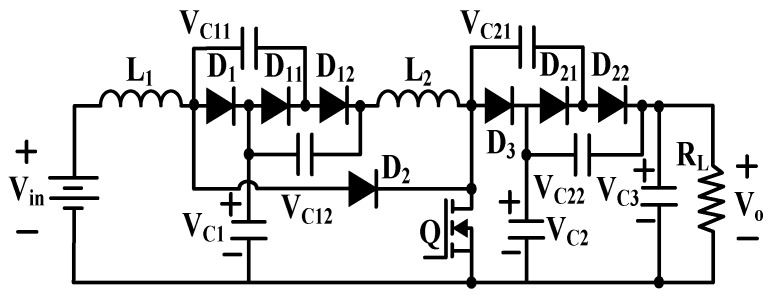
Two-stage cascade boost converter.

The cascade boost converter is suitable for driving piezoelectric actuators in hundred volt level. Nevertheless, DEAs are electrically actuated material devices that produce large deformation when a high driving voltage in a few thousand volts is applied to the electrode. The conversion between low voltage coming from lithium battery and high exciting voltage, which can drive DEA actuators, is not enough. Therefore, the conventional cascade boost converter becomes impractical. A feasible circuit that can achieve a few thousand volts with compact package [76] is presented in Figure 17. The two-stage cascade boost converter is derived from the two-stage boost converter by adding a double/enhanced circuit in each conversion stage.

**Figure 18:**
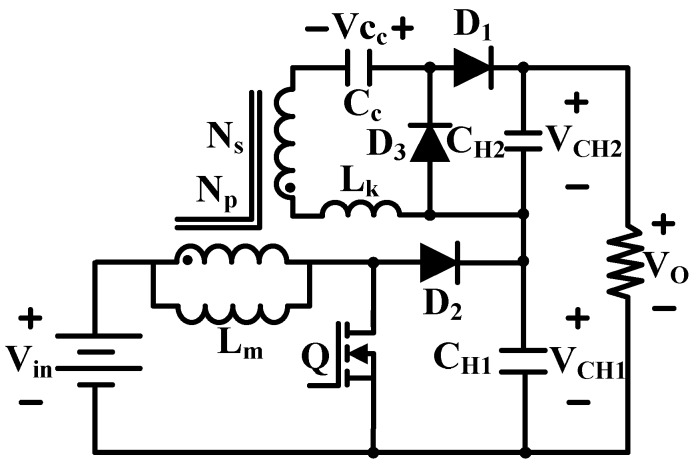
High conversion ratio boost converter.

Figure 18 shows the circuit configuration of high conversion ratio boost converter. This boost converter is used to transfer energy from the DC source V_in_ in the low-voltage side to the DC output V_o_ in the high-voltage side. When the proposed converter is operating in the boost mode, the circuit characteristic is cascaded by the boost converter and flyback converter with the voltage doubled [77,78].

**Figure 19:**
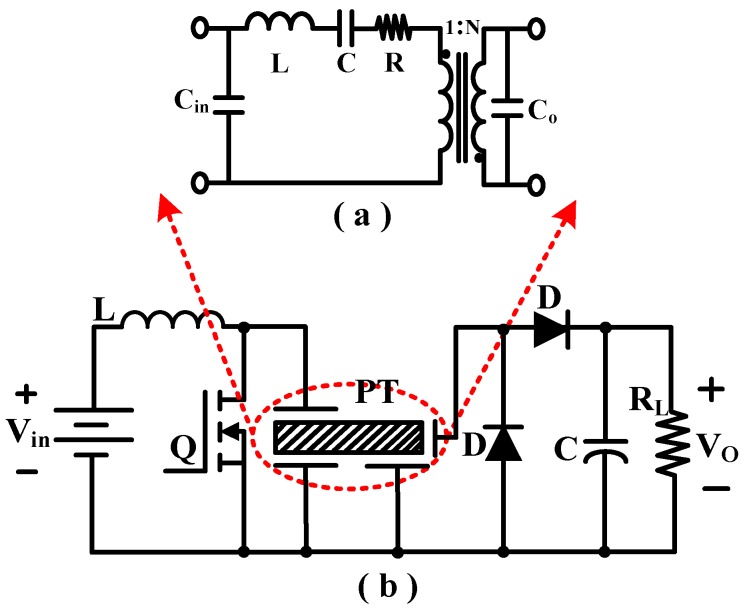
(**a**) Piezoelectric transformer equivalent circuit. (**b**) Class “E” power amplifier topology.

PTs have high voltage gain ratio and high power density (up to 40 W/cm^3^) [79] and have been widely used in actuators and sensors. Generally, PTs have to operate close to the mechanical resonance frequency to obtain high voltage gain and power efficiency. The equivalent electrical circuit of a PT is shown in Figure 19a. The gain of a PT is high at low loads, making it a good candidate for the high-voltage, low-current requirements of voltage-mode actuators. 

Figure 19b shows the class “E” resonant topology has a low number of additional components. The inductor is selected to resonate with the input capacitance C_in_ of the PT at a frequency close to the mechanical resonance frequency [80]. The resonance transfers energy to the PT from the inductor when the switch is off. The switch is turned on again as soon as the voltage across C_in_ is zero. Regulation of the output voltage is achieved by varying the switching frequency.

Table 3 shows some valuable parameters for comparing and quantifying the five types of topology performance parameters.

According to Table 3, a hybrid voltage multiplier boost converter has n times gain in contrast to a conventional boost converter because it uses n times voltage multiplier. The disadvantage of this topology is its large size, high weight and low efficiency (caused by the multiplier). However, it is commonly used in hundreds of voltage outputs because it is easily fabricated, with a tapped inductor boost converter capable of achieving the boosting capability without a high duty cycle. To achieve high voltage gains, this method has a considerably lesser number of parts than the hybrid voltage multiplier boost converter. However, the rectifier diode and output capacitor must be rated for the output voltage. Additionally, a custom transformer should be demanded to meet the low mass requirement in microrobotic applications because no commercial parts below 10 g can be purchased. As indicated in Table 3, an n-stage cascade boost converter has a higher gain than the traditional boost converter. The gain also depends on the switching duty cycle. With this simple converter, a high boosting function can be obtained. As illustrated in Table 3, a high conversion ratio boost converter can obtain high voltage gain by increasing the turns ratio of the coupled inductor. This converter has the same disadvantage as a tapped inductor boost converter. The customized transformer is a critical factor and difficult to fabricate. APT is better than a magnetic transformer because of its simple geometries, giving it potential in milligram-scale power actuator design.

### 5.3. Drive Stage

**Figure 20:**
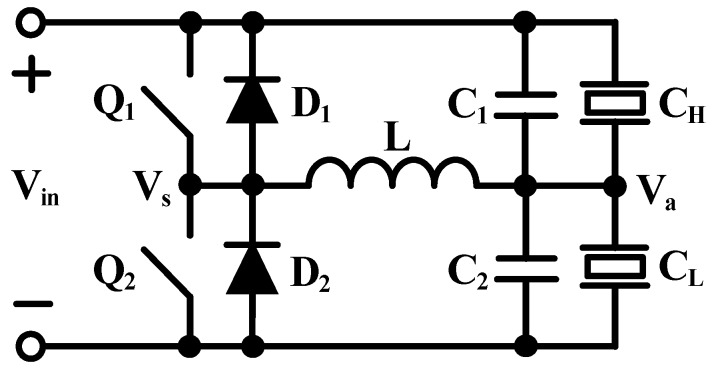
Switching amplifier driver with LC.

As mentioned earlier, these converters are DC–DC converters whose outputs are high enough to drive the piezoelectric actuator. If we want the wings of robotic insects to start vibrating, then an arbitrary unipolar drive voltage should be provided. Using an inductor, two additional switches and two self-timed shutdown diodes with capacitive loads, [81] proposed a highly remarkable energy recovery from the wing vibration. However, this proposed design only focuses on the charge recovery of piezoelectric actuators with quasi-square waves. Another feasible method is to use an LC resonance to obtain an arbitrary driving wave [4]. This topology is called the switching amplifier driver (Figure 20). 

After a series of charge and discharge pulses is applied to Q_1_ and Q_2_ at the appropriate time, an arbitrary waveform can be generated at V_a_. Differently, only a small amount of energy is processed in each switching cycle, which can be used to minimize the size of the inductor.

### 5.4. Control of Proposed Power Electronic Interfaces

**Figure 21:**
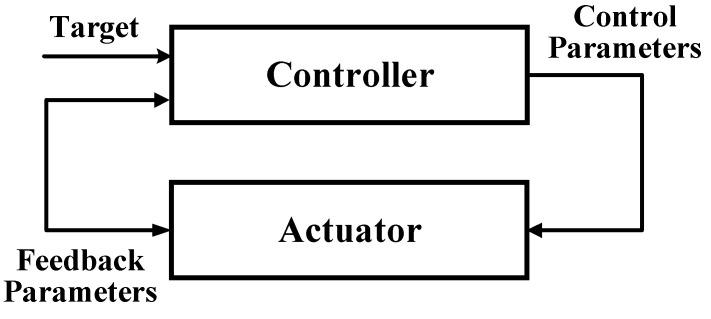
Block diagram of the control system.

After this the design and implementation of the proposed power electronic interfaces, a control system for the diagnostic of the actuator is needed and selected to be evaluated and implemented. The control system using estimation of the feedback parameters is shown in Figure 21.

The problem with the implementation of the control system is that it does not use the feedback of actuator displacement directly, instead it comes from the driving voltage/current estimation. The indirect feedback takes some time before the controller reduces/increase the control parameters when an overshoot/undershot occurs, if the estimated feedback parameters is too high or too low when a change is requested. To improve the controllability, one of the following techniques can be used (but is not limited to these).

Firstly, the Gain Scheduling Controller uses different control parameters depending either on the error, the size of the step or the region of the requested feedback parameters [82]. This should reduce the overshoot that some time when big steps are taken. One advantage of this is that the gain scheduling controller can handle the different regions of the actuator better than a conventional controller (static PI or PID controller, et al). But, the disadvantage is that is still needs to be tuned properly.

In addition, the LQ Controller uses a state space model of the actuator and an observer that is used to create the control signal to the system [83]. This requires both an accurate description of the system and observer. For the observer a Kalman filter is often used. One advantage is that the LQ controller both can handle disturbances and follow the reference signal equally good or better than conventional controller. The disadvantage is that the LQ controller is more complex to implement and tune than classic controller. However, if a good model of the actuator is available the implementation of the LQ controller becomes less complex.

The Self Tuning Controller is based on a black box model of the actuator [84]. To get an estimation of the black box model, the control system estimation needs to be done online. Then the controller uses parameters from the control system estimation to calculate the new control strategy. This will allow the control system to adapt to changes that occur due to differences in the load force and other external changes. One advantage of this is that it is capable of handle changes to the control system without losing the simplicity of the PID controller. Nevertheless, it needs to have a control system estimator that makes it hard to guarantee the stability of the control system because of the dynamics of the estimation.

The general solutions of the three described controllers can be used to control the described power electronic interfaces for actuators and even can be used in other applications. Self-Tuning Controller was chosen to be used more generally. One other reason was that it uses the PID structure which is more understandable than the Gain Scheduling Controller and LQ controller. 

## 6. Conclusions

This paper summarizes and discusses the system level of FWMAVs with a focus on state-of-the-art FWMAVs, aerodynamic mechanisms, transmission mechanisms and power electronic interfaces. First, various FWMAVs driven by electrical motor, mechanical transmission structure and “artificial muscles” material and investigated by research institutes are presented in detail. The unique aerodynamic modes of bird-mimetic flapping wing and insect-mimetic flapping wing aerial vehicles, which are unlike those of fixed-wing and rotary-wing aerial vehicles, are likewise elaborated. The selection and design of the mechanical transmission are considered based on the stringent requirement of physical and electrical performance in micrometer- to centimeter-scale level. Finally, power electronic topologies suitable for driving “artificial muscle” materials used in FWMAVs are stated. These results present some possible solutions for the creation of insect-sized FWMAVs and a substantial step toward the realization of flying microrobots. 

Further size and weight reductions of FWMAVs are important issues for the future. MEMS technologies can be used to provide devices, such as lighter, smaller and less power consuming components than the current state of-the-art ones. Nanotechnology could play an important role also in aerodynamic improvements. FWMAVs will most likely be equipped with GPS and radar systems. Infrared and/or high-definition cameras could be included. Furthermore, trends could also include the development of sophisticated software that will enable the operation of future ultrasmall FWMAVs. Finally, with the improvements of artificial intelligence, some of them will have decision-making capabilities, opening the way to completely new mission profiles.

## Figures and Tables

**Table 1:** Characteristics of the above FWMAVs.

Name/Manufacturer	Mass (g)	Wingspan (cm)	Flight Duration (min)
Microbat	12.5	25	0.7
Hummingbird	19	16.5	4
Phoenix	1200	-	-
H^2^bird	13.6	26.5	-
University of Arizona	248	74	7
University of Maryland	425	107	-
Robo Raven	690	150	15
Smart Bird	450	50	-
DelFly	21	50	-
DelFly Micro	3.07	10	-
Konkuk University	7.36	12.5	-
Bat Bot	93	30	-
Universite’ Libre de Bruxelles	22	21	0.3
Golden Snitch	8	20	5
Wasp AE	1300	108	50
Artificial Butterfly	-	-	A few seconds
Robobee (Harvard University)	0.08	3	-

**Table 2:** Overview characteristics of smart actuators [43].

Actuator Type	Maximum Strain (%)	Maximum Stress (MPa)	Specific Elastic Energy Density (J/g)	Maximum Efficiency (%)	Relative Speed
Dielectric elastomer (acrylic)	380	7.2	3.4	60–80	Medium
Dielectric elastomer (silicone)	63	3.0	0.75	90	Fast
Electrostatic	50	0.03	0.0015	>90	Fast
Electromagnetic	50	0.10	0.003	>90	Fast
Piezoelectric (ceramic)	0.2	110	0.013	90	Fast
Piezoelectric (single crystal)	1.7	131	0.13	90	Fast
Piezoelectric (polymer)	0.1	4.8	0.0013	80 est.	Fast
Shape memory alloy	>5	>200	>15	<10	Slow
Shape memory polymer	100	4	2	<10	Slow
Thermal polymer	15	78	0.15	<10	Slow
Electro-chemo-mechanical Conducting polymer	10	450	23	<5% est.	Slow
Mechanochemical polymer	>40	0.3	0.06	30	Slow

**Table 3:** Comparison of the main parameters of the five described topologies.

Components	Hybrid Voltage Multiplier	Tapped Inductor Boost Convertor	N-Stage Cascade Boost Converter	High Conversion Ratio Boost Converter	Class “E” Power Amplifier
Inductor	1	1	n	1	1
Capacitor	2n	1	n	3	1
Diode	2n + 1	1	n + 1	3	2
Switch	1	1	1	1	1
Gain	$\frac{n}{1-D}$	$\frac{DN+1}{1-D}$	${\left(\frac{1}{1-D}\right)}^{n}$	$\frac{N+1}{1-D}$	Fixed (≥100)

Note: *n* is the number of cascaded stages. *N* is the turns ratio between secondary side and primary side of the transformer.

## References

[1] Whitney J.P., Wood R.J. (2010). Aeromechanics of passive rotation in flapping flight. J. Fluid Mech..

[2] Li L., Wu Z. (2018). Research on automatic navigation of unmanned aerial vehicle based on 3D laser scanning. Laser J..

[3] Wood R.J., Steltz E., Fearing R.S. (2005). Optimal energy density piezoelectric bending actuators. Sens. Actuators A Phys..

[4] Karpelson M., Wei G., Wood R.J. Milligram-scale high-voltage power electronics for piezoelectric microrobots. Proceedings of the IEEE International Conference on Robotics and Automation.

[5] Pornsin-Sirirak T.N., Tai Y.C., Ho C.M., Keennon M. Microbat: A palm-sized electrically powered ornithopter. Proceedings of the NASA/JPL Workshop on Biomorphic Robotics.

[6] Keennon M., Klingebiel K., Won H. Development of the nano hummingbird: A tailless flapping wing micro air vehicle. Proceedings of the 50th AIAA Aerospace Sciences Meeting Including the New Horizons Forum and Aerospace Exposition.

[7] Li X. (2013). Laser Power Beaming for UAVs. Laser J..

[8] Rose C., Fearing R.S. Comparison of ornithopter wind tunnel force measurements with free flight. Proceedings of the IEEE International Conference on Robotics and Automation.

[9] Krashanitsa R.Y., Silin D., Shkarayev S.V., Abate G. (2009). Flight dynamics of a flapping-wing air vehicle. Int. J. Micro Air Veh..

[10] Liu F., Lin J., Wang Y., Chen C. (2014). The design of High Precision QCL Driver for Micro Laser Impulsed Unmanned Aerial Vehicle. Laser J..

[11] Gerdes J., Holness A., Perez-Rosado A., Roberts L., Greisinger A., Barnett E., Kempny J., Lingam D., Yeh C.H., Bruck H.A. (2014). Robo Raven: A flapping-wing air vehicle with highly compliant and independently controlled wings. Soft Robot..

[12] Mackenzie D. (2012). A flapping of wings. Science.

[13] Tijmons S., De Wagter C., Remes B., de Croon G. (2018). Autonomous Door and Corridor Traversal with a 20-Gram Flapping Wing MAV by Onboard Stereo Vision. Aerospace.

[14] De Croon G.C., Groen M.A., De Wagter C., Remes B., Ruijsink R., Van Oudheusden B.W. (2012). Design, aerodynamics and autonomy of the DelFly. Bioinspir. Biomim..

[15] Karásek M., Muijres F.T., De Wagter C., Remes B.D., de Croon G.C. (2018). A tailless aerial robotic flapper reveals that flies use torque coupling in rapid banked turns. Science.

[16] De Croon G.C.H.E., Perçin M., Remes B.D.W., Ruijsink R., De Wagter C. (2016). The DelFly: Design, Aerodynamics, and Artificial Intelligence of a Flapping Wing Robot.

[17] Phan H.V., Truong Q.T., Park H.C. (2015). Implementation of initial passive stability in insect-mimicking flapping-wing micro air vehicle. Int. J. Intell. Unmanned Syst..

[18] Ramezani A., Chung S.J., Hutchinson S. (2017). A biomimetic robotic platform to study flight specializations of bats. Sci. Robot..

[19] Roshanbin A., Altartouri H., Karásek M., Preumont A. (2017). COLIBRI: A hovering flapping twin-wing robot. Int. J. Micro Air Veh..

[20] Hsiao F.Y., Yang L.J., Lin S.H., Chen C.L., Shen J.F. (2012). Autopilots for ultra lightweight robotic birds: Automatic altitude control and system integration of a sub-10 g weight flapping-wing micro air vehicle. IEEE Control Syst..

[21] Hamilton S.L. (2012). UAVs: Unmanned Aerial Vehicles.

[22] Tanaka H., Shimoyama I. (2010). Forward flight of swallowtail butterfly with simple flapping motion. Bioinspir. Biomim..

[23] Sahai R., Galloway K.C., Wood R.J. (2013). Elastic element integration for improved flapping-wing micro air vehicle performance. IEEE Trans. Robot..

[24] Hines L., Campolo D., Sitti M. (2014). Liftoff of a motor-driven, flapping-wing microaerial vehicle capable of resonance. IEEE Trans. Robot..

[25] Yuk H., Shin J.H., Jo S. Design and control of thermal SMA based small crawling robot mimicking *C. elegans*. Proceedings of the IEEE/RSJ International Conference on Intelligent Robots and Systems.

[26] Gilpin K., Torres-Jara E., Rus D. (2007). Controlling Closed-Chain Robots with Compliant SMA Actuators: Algorithms and Experiments.

[27] Chen C., Tang Y., Khaligh A., Newcomb R.W. A Low-power and High-gain Converter for Driving Dielectric Elastomer Actuators. Proceedings of the Twenty-Eighth Annual IEEE Applied Power Electronics Conference and Exposition.

[28] Jung K., Koo J.C., Lee Y.K., Choi H.R. (2007). Artificial annelid robot driven by soft actuators. Bioinspir. Biomim..

[29] Steltz E., Avadhanula S., Fearing R.S. High Lift Force with 275 Hz Wing Beat in MFI. Proceedings of the IEEE/RSJ International Conference on Intelligent Robots and Systems.

[30] Jayaram K., Jafferis N., Doshi N., Goldberg B., Wood R.J. (2018). Concomitant Sensing and Actuation for Piezoelectric Microrobots. Smart Mater..

[31] Wood R.J. Liftoff of a 60mg flapping-wing MAV. Proceedings of the IEEE/RSJ International Conference on Intelligent Robots and Systems.

[32] Ma K.Y., Chirarattananon P., Fuller S.B., Wood R.J. (2013). Controlled flight of a biologically inspired, insect-scale robot. Science.

[33] Van den Berg C., Ellington C.P. (1997). The vortex wake of a ‘hovering’model hawkmoth. Philos. Trans. R. Soc. B Biol. Sci..

[34] Liu H., Ellington C.P., Kawachi K., Van Den Berg C., Willmott A.P. (1998). A computational fluid dynamic study of hawkmoth hovering. J. Exp. Biol..

[35] Weis-Fogh T. (1975). Unusual mechanisms for the generation of lift in flying animals. Sci. Am..

[36] Dickinson M. (1994). The effects of wing rotation on unsteady aerodynamic performance at low Reynolds numbers. J. Exp. Biol..

[37] Dickinson M.H., Gotz K.G. (1993). Unsteady aerodynamic performance of model wings at low Reynolds numbers. J. Exp. Biol..

[38] Dickinson M.H., Lehmann F.O., Sane S.P. (1999). Wing rotation and the aerodynamic basis of insect flight. Science.

[39] Gardano P., Dabnichki P. (2006). Application of boundary element method to modelling of added mass and its effect on hydrodynamic forces. CMES Comput. Model. Eng. Sci..

[40] Andro J.Y., Jacquin L. (2009). Frequency effects on the aerodynamic mechanisms of a heaving airfoil in a forward flight configuration. Aerosp. Sci. Technol..

[41] Giesing J.P. (1968). Nonlinear two-dimensional unsteady potential flow with lift. J. Aircr..

[42] Carpi F., Roy K., Peter S., Gursel A. (2011). Electroactive polymer actuators as artificial muscles: Are they ready for bioinspired applications?. Bioinspir. Biomim..

[43] Kornbluh R.D., Pelrine R., Pei Q., Heydt R., Stanford S., Oh S., Eckerle J. Electroelastomers: Applications of dielectric elastomer transducers for actuation, generation, and smart structures. Proceedings of the SPIE’s 9th Annual International Symposium on Smart Structures and Materials.

[44] Yoichi M. (2006). Applications of piezoelectric actuator. NEC Tech. J..

[45] Karpelson M., Wei G.Y., Wood R.J. (2012). Driving high voltage piezoelectric actuators in microrobotic applications. Sens. Actuators A Phys..

[46] Roll J.A., Cheng B., Deng X. (2015). An electromagnetic actuator for high-frequency flapping-wing microair vehicles. IEEE Trans. Robot..

[47] Pang G., Deng J., Wang F., Zhang J., Pang Z., Yang G. (2018). Development of Flexible Robot Skin for Safe and Natural Human–Robot Collaboration. Micromachines.

[48] Wood R.J. (2008). The first takeoff of a biologically inspired at-scale robotic insect. IEEE Trans. Robot..

[49] Feng L., Zhou Q., Song B., Feng Y., Cai J., Jiang Y., Zhang D. (2018). Cell Injection Millirobot Development and Evaluation in Microfluidic Chip. Micromachines.

[50] Sreetharan P.S., Wood R.J. (2011). Passive torque regulation in an underactuated flapping wing robotic insect. Auton. Robot..

[51] Pérez-Arancibia N.O., Ma K.Y., Galloway K.C., Greenberg J.D., Wood R.J. (2011). First controlled vertical flight of a biologically inspired microrobot. Bioinspir. Biomim..

[52] Lok M., Brooks D., Wood R., Wei G.Y. Design and analysis of an integrated driver for piezoelectric actuators. Proceedings of the IEEE Energy Conversion Congress and Exposition.

[53] Lok M., Zhang X., Helbling E.F., Wood R., Brooks D., Wei G.Y. A power electronics unit to drive piezoelectric actuators for flying microrobots. Proceedings of the IEEE Custom Integrated Circuits Conference.

[54] Li Q., Hao X., He J., Li X., Wang J., Li H. (2016). The Influences of Refractive Index Sensing of Metamaterials with Different Size of Dielectric Layer. Laser J..

[55] Madden J.D., Vandesteeg N.A., Anquetil P.A., Madden P.G., Takshi A., Pytel R.Z., Hunter I.W. (2004). Artificial muscle technology: Physical principles and naval prospects. IEEE J. Ocean. Eng..

[56] Shintake J., Rosset S., Schubert B., Mintchev S., Floreano D., Shea H. DEA for soft robotics: 1-gram actuator picks up a 60-gram egg. Proceedings of the SPIE’s Smart Structures and Materials + Nondestructive Evaluation and Health Monitoring.

[57] Nicholas S., Baker M.H., Daniel S., Ryan D. Design of a Flapping Wing Micro Air Vehicle Actuation System. Proceedings of the 2012 ASEE North-Central Section Conference.

[58] Bronson J.R., Pulskamp J.S., Polcawich R.G., Kroninger C.M., Wetzel E.D. PZT MEMS actuated flapping wings for insect-inspired robotics. Proceedings of the IEEE 22nd International Conference on Micro Electro Mechanical Systems.

[59] Tang Y., Chen C., Khaligh A., Penskiy I., Bergbreiter S. (2014). An ultracompact dual-stage converter for driving electrostatic actuators in mobile microrobots. IEEE Trans. Power Electron..

[60] Meng K., Zhang W., Chen W., Li H., Chi P., Zou C., Chen J. (2012). The design and micromachining of an electromagnetic MEMS flapping-wing micro air vehicle. Microsyst. Technol..

[61] Roll J.A., Cheng B., Deng X. Design, fabrication, and experiments of an electromagnetic actuator for flapping wing micro air vehicles. Proceedings of the IEEE International Conference on Robotics and Automation.

[62] Cheng B., Roll J.A., Deng X. Modeling and optimization of an electromagnetic actuator for flapping wing micro air vehicle. Proceedings of the IEEE International Conference on Robotics and Automation.

[63] Schneuwly A. (2005). Charge ahead. Power Eng..

[64] Bellew C.L., Hollar S., Pister K.S.J. An SOI Process for Fabrication of Solar Cells, Transistors, and Electrostatic Actuators. Proceedings of the 12th International Conference on Solid State Sensors, Actuators and Microsystems.

[65] Wilhelm A., Surgenor B.W., Pharoah J.G. Evaluation of a Micro Fuel Cell as Applied to a Mobile Robot. Proceedings of the IEEE International Confernce on Mecharonics and Automation.

[66] Ortiz-Lopez M.G., Leyva-Ramos J.E., Carbajal-Gutierrez E., Morales-Saldana J.A. (2008). Modelling and analysis of switch-mode cascade converters with a single active switch. IET Power Electron..

[67] Lin W., Wang J., Huang J., Xu Y. A Novel Tapped Inductor Bi-directional Buck-Boost Topology. Proceedings of the 30th International Telecommunications Energy.

[68] Li Q., Hao X., Wang J., Li X., Li H., Li H., He J. (2017). Metamaterials-based tunable perfect absorbers. Laser J..

[69] Karpelson M., Wei G., Wood R.J. A Review of Actuation and Power Electronics Options for Flapping-Wing Robotic Insects. Proceedings of the IEEE International Conference on Robotics and Automation.

[70] Steltz E., Seeman M., Avadhanula S., Fearing R.S. Power Electronics Design Choice for Piezoelectric Microrobots. Proceedings of the International Conference on Intelligent Robots and Systems.

[71] Saheb J.F., Richard J.F., Sawan M., Meingan R., Savaria Y. (2007). System integration of high voltage electrostatic MEMS actuators. Analog Integr. Circuits and Signal Process..

[72] Chen C., Liu M., Wang Y. (2018). A Dual Stage Low Power Converter Driving for Piezoelectric Actuator Applied in Micro Mobile Robot. Appl. Sci..

[73] Luo F.L. (1999). Negative Output Luo-Converters, Voltage Lift Technique. IET Electr. Power.

[74] Luo F., Ye H., Rashid M.H. (2002). Multiple-quadrant Luo-converters. IEE Proc. Electr. Power Appl..

[75] Qin A. (2017). Efficient application research on high frequency switching power supply. Laser J..

[76] Luo F.L., Ye H. (2004). Positive output cascade boost converters. IEE Proc. Electr. Power Appl..

[77] Chen T.M., Chen C.L. (2002). Analysis and design of asymmetrical half bridge flyback converter. IEE Proc. Electr. Power Appl..

[78] Jeong G.Y. (2010). High efficiency asymmetrical half-bridge flyback converter using a new voltage-driven synchronous rectifier. IET Power Electron..

[79] Chen C., Wang Y. (2013). A review of fabrication options and power electronics for flapping-wing robotic insects. Int. J. Adv. Robot. Syst..

[80] Dallago E., Danioni A., Ricotti G., Venchi G. Single chip, Low Supply Voltage Piezoelectric Transformer Controller. Proceedings of the 29th European Solid-State Circuits Conference.

[81] Campolo D., Sitti M., Fearing R.S. (2003). Efficient charge recovery method for driving piezoelectric actuators with quasi-square waves. IEEE Trans. Ultrason. Ferroelectr. Freq. Control.

[82] Gahinet P., Arkadii N., Alan J.L., Mahmoud C. The LMI control toolbox. Proceedings of the 33th IEEE Conference on Decision and Control.

[83] Bouabdallah S., Andre N., Roland S. PID vs LQ control techniques applied to an indoor micro quadrotor. Proceedings of the IEEE International Conference on Intelligent Robots and Systems.

[84] Clarke D.W., Peter J.G. (1975). Self-tuning controller. Proc. Inst. Electr. Eng..

